# Anti-Inflammatory, Wound Healing, and Anti-Diabetic Effects of Pure Active Compounds Present in the Ryudai Gold Variety of *Curcuma longa*

**DOI:** 10.3390/molecules29122795

**Published:** 2024-06-12

**Authors:** Md Zahorul Islam, Jesmin Akter, Md Amzad Hossain, Md Shafiqul Islam, Purba Islam, Chayon Goswami, Ha Thi Thanh Nguyen, Atsushi Miyamoto

**Affiliations:** 1 Department of Pharmacology, Faculty of Veterinary Science, Bangladesh Agricultural University, Mymensingh 2202, Bangladesh; shafiqpharma@yahoo.co.uk (M.S.I.); purba.islam@bau.edu.bd (P.I.); 2 Faculty of Agriculture, University of the Ryukyus, Okinawa 903-0213, Japan; jesminbau02@gmail.com; 3 The United Graduate School of Agricultural Sciences, Kagoshima University, Kagoshima 890-0065, Japan; 4 Department of Biochemistry and Molecular Biology, Faculty of Agriculture, Bangladesh Agricultural University, Mymensingh 2202, Bangladesh; chayon.goswami@bau.edu.bd; 5 Department of Veterinary Pharmacology, Faculty of Veterinary Medicine, Vietnam National University of Agriculture, Trau Quy Crossing, Gia Lam District, Hanoi 131000, Vietnam; 650537@sv.vnua.edu.vn; 6 Department of Veterinary Pharmacology, Joint Faculty of Veterinary Medicine, Kagoshima University, 1-21-24 Korimoto, Kagoshima 890-0065, Japan; k1330977@kadai.jp

**Keywords:** curcumin, demethoxycurcumin, bisdemethoxycurcumin, diabetes, inflammation, wound

## Abstract

Turmeric (*Curcuma longa*) contains curcumin, demethoxycurcumin (DMC), and bisdemethoxycurcumin (BDMC). Nevertheless, curcumin is the most researched active ingredient for its numerous pharmacological effects. We investigated the impact of these curcuminoids found in Ryudai gold, an approved cultivar of *Curcuma longa*, on wound healing, inflammation, and diabetes. Sub-planter injections of carrageenan induced acute paw inflammation in rats. The wound-healing ability of 1% curcuminoids was examined by making a 6 mm round wound on the shaved dorsum of the mice with a biopsy punch. A single intraperitoneal injection of streptozotocin (50 mg/kg) was used to induce diabetes in mice. Curcuminoids at a dose rate of 100 mg/kg body weight were used with feed and as a gastric gavage to treat diabetes and inflammation in experimental animals. Paw thickness was measured at 1, 3, and 6 h following carrageenan injection. After three hours, mean paw volume was 58% in carrageenan-injected mice, which was 35%, 37%, and 31% in the curcumin, DMC, and BDMC groups, respectively. Histopathology of the paw tissue demonstrated severe infiltration of inflammatory cells and thickening of the dermis, which were remarkably improved by the curcuminoids. The wound-healing abilities were significantly higher in the curcumin- (95.0%), DMC- (93.17%), and BDMC-treated (89.0%) groups, in comparison to that of the control (65.09%) group at day nine. There were no significant differences in wound-healing activity among the groups treated with 1% curcuminoids throughout the study. Streptozotocin-induced diabetes was characterized by an increased blood glucose (552.2 mg/dL) and decreased body weight (31.2 g), compared to that of the control rats (145.6 mg/dL and 46.8 g blood glucose and body weight, respectively). It also caused an increase in serum alanine aminotransferase (ALT; 44.2 U/L) and aspartate aminotransferase (AST; 55.8 U/L) compared to that of the control group (18.6 U/L and 20.1 U/L, respectively). Histopathological examination of the liver showed that diabetes caused hepatic cellular necrosis, congestion of the central vein, and parenchymatous degeneration. However, all three curcuminoids significantly decreased blood glucose levels, ALT, and AST and improved the histopathological score of the liver. These results evidenced that not only curcumin but also DMC and BDMC have potent anti-inflammatory, wound healing, and anti-diabetic efficacy, and the Ryudai gold variety of turmeric could be used as a functional food supplement.

## 1. Introduction

**Figure 1:**
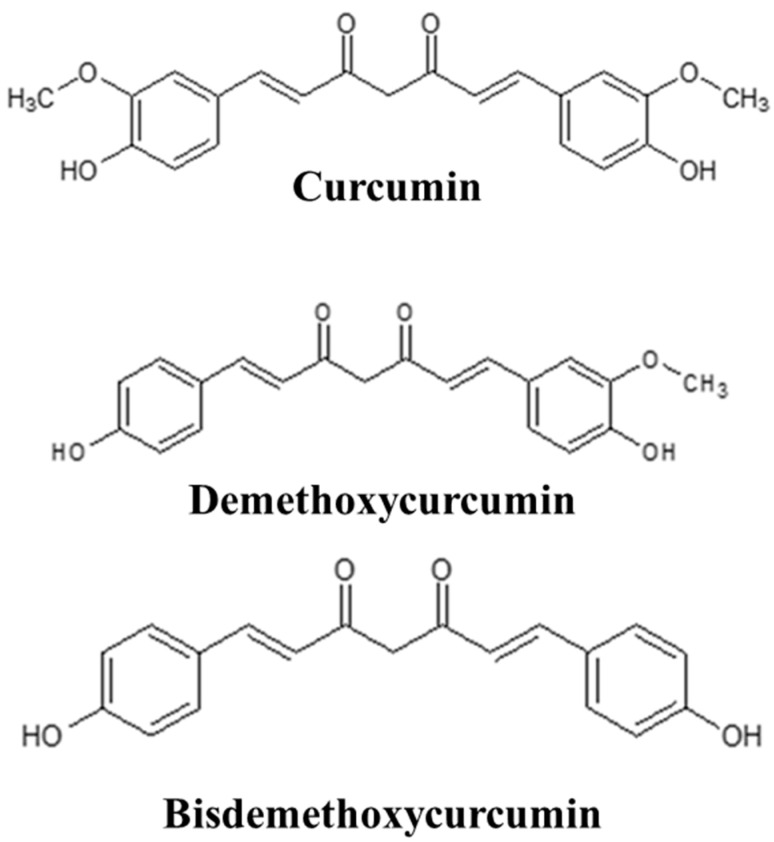
Chemical structure of curcumin, demethoxycurcumin, and bisdemethoxycurcumin.

The rhizome of *Curcuma longa* (turmeric) is commonly utilized in traditional medicine. Extensive studies have been conducted to ascertain the biological activity of turmeric extract. Since curcumin is the primary bioactive molecule of turmeric, researchers are primarily interested in studying it among the active ingredients. However, chromatographic analysis revealed that *Curcuma longa* always contains curcumin, demethoxycurcumin (DMC), and bisdemethoxycurcumin (BDMC) [1]. A comparative study on the curcuminoid content of different species and varieties of turmeric was reported in our paper previously [2]. We also isolated and purified curcumin, DMC, and BDMC along with other compounds from Ryudai gold, a new variety (registration no. 21485, 2012; Ministry of Agriculture, Forestry and Fisheries, Japan) of *Curcuma longa* (Figure 1). This cultivar contains higher curcumin, DMC, and BDMC concentrations with stronger antifungal [2,3] and antioxidant [4] activities. Curcumin has a low oral bioavailability due to its poor solubility in water and instability in an acidic pH. It is also rapidly metabolized and cleared from the body. Conversely, DMC and BDMC exhibit superior chemical stability and bioavailability at a physiological pH compared to curcumin. The latter two are the most abundant curcuminoids after curcumin, and their functions, in some cases, are better than curcumin [5]. Thus, in cases where curcumin is ineffective, these two chemicals can be utilized as substitutes.

Inflammation is a self-defense strategy to initiate the healing process. Numerous diseases, including diabetes, arthritis, atherosclerosis, Alzheimer’s disease, neurodegeneration, and cancer, are influenced by chronic inflammation. Thus, research on the anti-inflammatory mechanism may offer a novel strategy for reducing the inflammatory consequences of diabetes and promoting wound healing. Turmeric extract exhibits anti-inflammatory characteristics in response to dimethylbenzene-induced ear inflammation in mice and carrageenan-induced paw edema in a rat model [6]. Like indomethacin, turmeric oil inhibits inflammatory prostaglandin E_2_ production via the inhibition of COX-2 [7]. Curcumin-dependent and -independent anti-inflammatory activities of turmeric have also been reported [8]. Nevertheless, little research has been carried out on the anti-inflammatory mechanisms of DMC and BDMC.

Wound healing is the process of repairing injured tissues. Turmeric powder application is one of the traditional and local treatments that some people, particularly those in the elderly age group, still prefer to utilize instead of seeking medical facilities. It has been reported that the crude extract of turmeric increased the proliferation of cells and the synthesis of collagen fiber at the wound sites in normal rats [9]. Fresh turmeric paste also significantly promoted wound healing in a rabbit model [10].

Diabetes is a lifestyle disease with complications including hypertension, delayed wound healing, neuropathy, and tissue hypoxia [11]. Turmeric is a well-recognized ayurvedic medicine for diabetes [12]. We previously documented the anti-diabetic properties of a novel molecule isolated from *Curcuma longa* [13]. Additionally, earlier research demonstrated that curcuminoids and sesquiterpenoids from *Curcuma longa* could effectively lower blood glucose levels in KK-Ay mice with type 2 diabetes [14]. According to Sidhu et al., DMC and BDMC are promising components of functional foods that improve tissue remodeling to heal diabetic wounds [15]. Retinoic acid, aspirin, resveratrol, and curcumin have been reported to prevent diabetic nephropathy and cardiomyopathy through an anti-inflammatory mechanism [16,17,18,19]. Hence, targeting inflammatory pathways could be a significant strategy for treating diabetes [20,21].

Diabetes, inflammation, and sometimes wounds are chronic conditions usually managed with long-term therapy, which is difficult to follow and costs a lot of money, with some adverse effects. Therefore, natural ingredients gained research focus as an alternative to conventional therapeutic strategies for these conditions. Turmeric is a relatively cheap, ubiquitous, and available spice in this subcontinent. It is traditionally used for many diseases. The precise chemical exhibiting anti-inflammatory, wound healing, and anti-diabetic properties has yet to be determined. We have studied the antioxidant and antifungal potentials of active molecules isolated from the Ryudai gold variety of *Curcuma longa*, which has better activity and higher curcumin, DMC, and BDMC contents.

## 2. Results

### 2.1. Effects of Curcumin, Demethoxycurcumin, and Bisdemethoxycurcumin on Carrageenan-Induced Paw Edema in Rats

**Figure 2:**
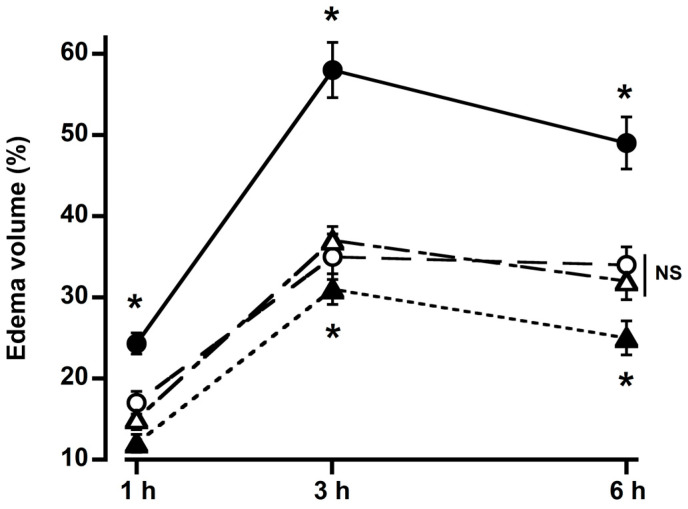
Effects of 100 mg/kg body weight of curcumin (○), demethoxycurcumin (△), and bisdemethoxycurcumin (▲) on rat paw edema caused by carrageenan (●). All three active compounds of turmeric significantly inhibit carrageenan-induced paw edema. Bisdemethoxycurcumin was more potent than the other two compounds. For each point, the mean ± SD of four rats is used. * *p* < 0.05 vs. carrageenan. NS = not significant.

The paw volume rose with the carrageenan injection. Treatment with curcumin, DMC, and BDMC (100 mg/kg bwt) significantly reduced edema size (Figure 2). Paw size reached its maximum (58%) after three hours of carrageenan injection, which was 35%, 37%, and 31% in the curcumin, DMC, and BDMC groups. Three hours following the carrageenan injection, all curcuminoids showed the most significant reduction in edema. The rate of inhibition of edema volume was low at 6 h. BDMC showed significantly more potent inhibition of edema among the curcuminoids. No discernible differences were found between the curcumin and DMC groups (Figure 2).

### 2.2. Histopathological Analysis of Rat Paw Tissue

**Figure 3:**
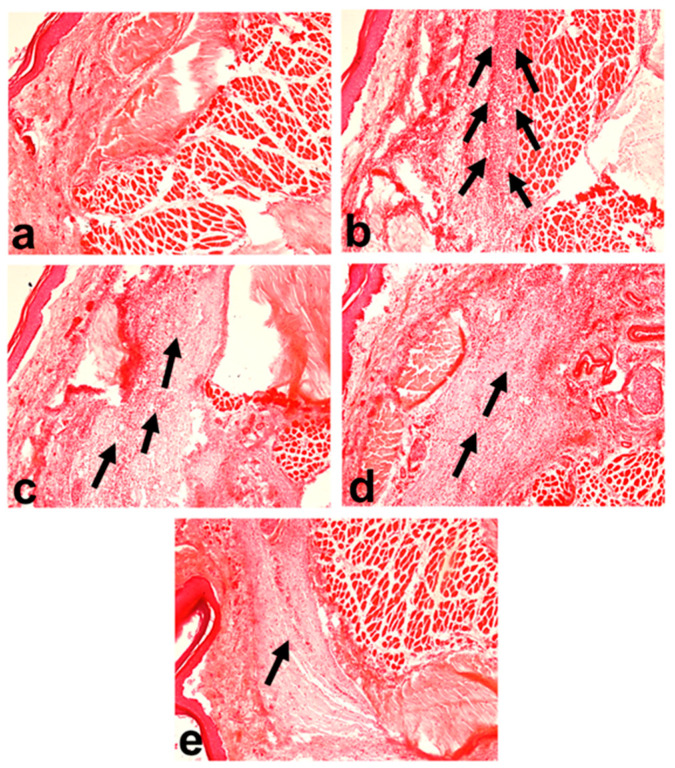
Histoarchitecture of rat paw tissue at 3 h of carrageenan injection (hematoxylin and eosin stain) (original magnification = 100× and scale bar = 50 μm): (**a**) control, showing normal architecture of dermis and hypodermis; (**b**) carrageenan, characterized by acute inflammation in the dermis and hypodermis extending to the underlying muscular tissue with a large influx of inflammatory cells; (**c**) carrageenan + curcumin; (**d**) carrageenan + demethoxycurcumin; and (**e**) carrageenan + bisdemethoxycurcumin showed a moderate density of inflammatory cells. The number of arrows shows the relative density of penetrated inflammatory cells.

The control group’s paw tissue showed a typical cellular structure with no signs of inflammation (Figure 3a). In contrast, the group treated with carrageenan showed an extensive influx of inflammatory cells with edema, thickening of the dermis, and distortion of the tissue’s architecture (Figure 3b). Treatment with curcumin, DMC, and BDMC (100 mg/kg but) markedly decreased the thickening of the dermis and epidermis, and the infiltration of inflammatory cells in the tissues of the rat paw at 3 h (Figure 3c–e).

### 2.3. Effects of Curcumin, Demethoxycurcumin, and Bisdemethoxycurcumin on Wound Healing

**Figure 4:**
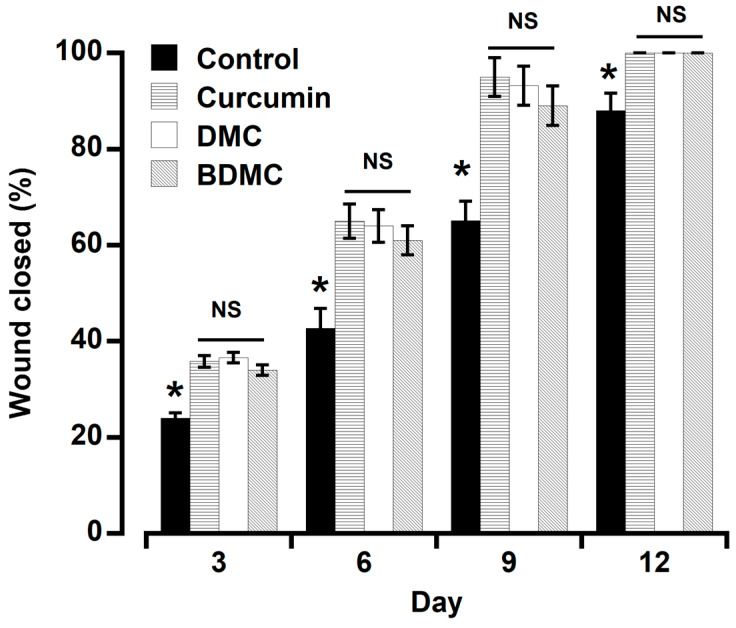
Effects of topical application of curcumin, DMC, and BDMC (1%) on the wound-healing time in mice. All the active compounds significantly accelerated the healing time. The three compounds did not differ substantially from one another. For each point, the mean ± SD of three mice is used. * *p* < 0.05 vs. curcuminoids. NS = not significant.

The wound closing rate was significantly higher in all the treatment groups throughout the period (Figure 4). On day six, 42.6% of the wounds were closed in the control group, and 65.0%, 63.98%, and 61.0% in the curcumin, DMC, and BDMC groups. Throughout the investigation, there was no discernible difference among the treated groups. On day 12, the macroscopic evaluation confirmed that the area of the wound in all treatment groups was fully closed (100%), except in the control group (88%).

### 2.4. Anti-Diabetic Study

#### 2.4.1. Effects of Curcumin, Demethoxycurcumin, and Bisdemethoxycurcumin on Body Weight

**Figure 5:**
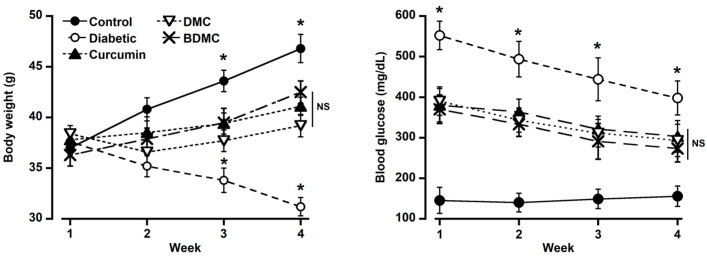
Effects of curcumin, demethoxycurcumin, and bisdemethoxycurcumin on diabetes-induced changes in body weight and blood glucose level in mice. Streptozotocin-induced diabetes significantly decreased body weight and increased blood glucose levels, which were significantly improved by all the compounds in turmeric. There was no significant difference among the three compounds. For each point, the mean ± SD of five mice is used. * *p* < 0.05 vs. control. NS = not significant.

The body weight was increased in the control group throughout the experiment but decreased significantly in the diabetic group (Figure 5; left panel). Treatment with curcumin, DMC, and BDMC at 100 mg/kg bwt considerably improved body weight loss due to diabetes caused by streptozotocin. There was no significant difference among the three treatment groups, but they differed significantly from the control and diabetic groups (Figure 5; left panel).

#### 2.4.2. Effects of Curcumin, Demethoxycurcumin, and Bisdemethoxycurcumin on Blood Glucose

The blood glucose level of the streptozotocin-injected group was significantly higher than that of the control group (Figure 5; right panel). The blood glucose level was in a decreasing pattern throughout the experiment in the diabetic group. However, it maintained a significantly higher blood glucose concentration than the other groups. Curcumin, DMC, and BDMC at 100 mg/kg bwt significantly decreased blood glucose levels. BDMC showed a more substantial glucose-lowering effect than curcumin and DMC. No difference was observed between curcumin- and DMC-treated groups (Figure 5; right panel). The glucose-lowering activity was decreased in the order of BDMC > curcumin > DMC.

#### 2.4.3. Effects of Curcumin, Demethoxycurcumin, and Bisdemethoxycurcumin on Blood Biochemical Parameters

**Figure 6:**
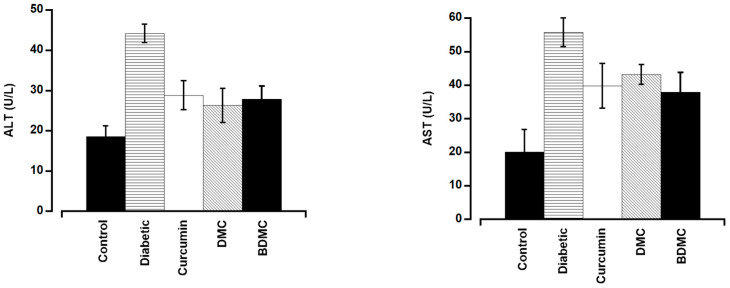
Curcumin, demethoxycurcumin, and bisdemethoxycurcumin affect serum ALT and AST levels in mice. The turmeric components significantly decreased blood ALT and AST levels in diabetic mice. There was no significant difference among the three compounds. For each point, the mean ± SD of five mice is used.

Serum ALT and AST levels were significantly greater in the group that received the intraperitoneal injection of streptozotocin (44.2 U/L and 55.8 U/L, respectively) than in the control group (18.6 U/L and 20.1 U/L, respectively), suggesting that the injection caused liver damage in the diabetic group (Figure 6). AST and ALT levels were significantly lower in the curcumin-, DMC-, and BDMC-treated groups than those of the diabetic group but higher than those of the control group (Figure 6).

#### 2.4.4. Effects of Curcumin, Demethoxycurcumin, and Bisdemethoxycurcumin on Liver Histopathology

**Figure 7:**
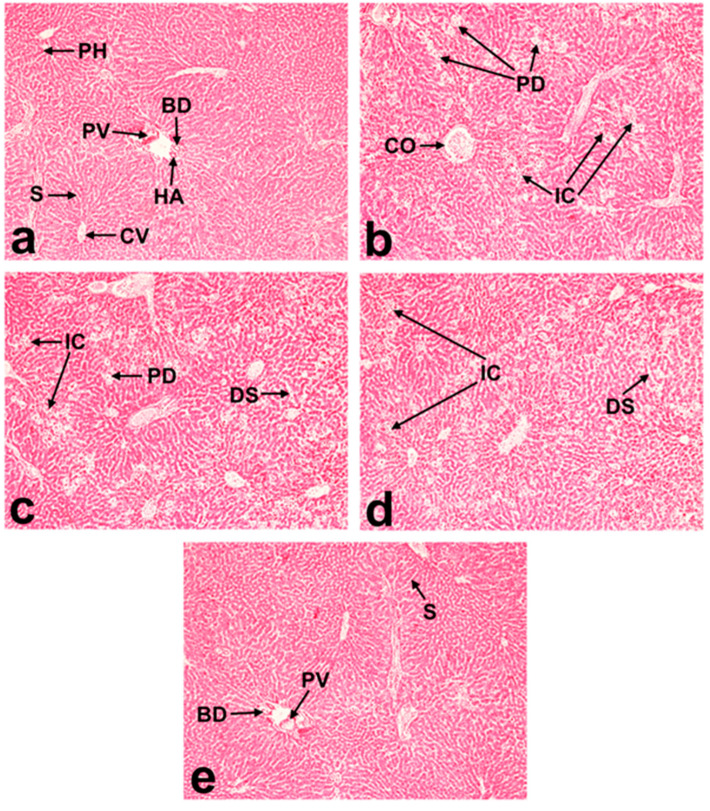
Effects of streptozotocin-induced diabetes on the histoarchitecture of mice livers (H&E staining): (**a**) control; (**b**) streptozotocin; (**c**) streptozotocin + curcumin; (**d**) streptozotocin + demethoxycurcumin; and (**e**) streptozotocin + bisdemethoxycurcumin; (magnification = 100×; scale bar = 50 μm). Arrows denote dilated sinusoid (DS), congested central vein (CO), parenchymatous degeneration (PD), polygonal hepatocytes (PH), portal vein (PV), central vein (CV), sinusoids (S), hepatic artery (HA), bile duct (BD), and mononuclear inflammatory cells (IC).

The hepatic tissue architecture in the control group was normal (Figure 7a). The streptozotocin-induced diabetic group showed dilation and congestion of the central vein, centrilobular necrosis, hepatocellular degeneration, and dispersed inflammatory cell invasion (Figure 7b). Treatment with curcumin, DMC, and BDMC (100 mg/kg bwt) markedly improved the streptozotocin-induced hepatic lesions (Figure 7c–e).

## 3. Discussion

The crude extracts of turmeric and curcumin have been reported to have anti-inflammatory, wound-healing, and anti-diabetic properties. The Ryudai gold cultivar of *Curcuma longa* contains a higher concentration of these curcuminoids [2] and shows better antifungal and antioxidant activities than other species of turmeric [3,4]. In an animal model, we compared the anti-inflammatory, wound-healing, and anti-diabetic activities of curcumin, DMC, and BDMC present in Ryudai gold.

Carrageenan-induced hind-paw edema is an extensively utilized primary screening test for the anti-inflammatory properties of organic and synthesized medicines [22]. In this investigation, the paw’s volume peaked (58%) three hours after the carrageenan injection. Curcumin, DMC, and BDMC (100 mg/kg bwt) pretreatment significantly decreased the edema caused by carrageenan. Among the three curcuminoids, BDMC showed the highest anti-inflammatory potency. This might be due to their differences in chemical structure, stability, and/or bioavailability. DMC and BDMC are more bioavailable and water-soluble than curcumin [23]. It has been reported that BDMC showed more inhibitory effects on COX-1 and COX-2 compared to curcumin and DMC [24]. All three curcuminoids demonstrated vigorous anti-inflammatory activity at 3 h after carrageenan injection. However, the inhibitory effects of the curcuminoids started decreasing after 3 h, which might be due to the metabolism of these compounds and their excretion from the body [25]. In a cell culture study, Guo et al. reported that DMC and BDMC showed anti-inflammatory action by inhibiting LPS-induced NF-kB activation [26]. Another study observed that DMC and BDMC are more potent anti-inflammatory agents but are less antioxidative than curcumin [23]. Gouthamchandra et al. reported that turmeric extract with enriched BDMC reduced rat paw edema by inhibiting different inflammatory mediators [27].

Histopathological analysis of the inflamed tissue demonstrated a higher number of inflammatory cells as well as thickening of the epidermis and dermis layers in the carrageenan-treated group. The number of inflammatory cells and thickening of the epidermis and dermis were effectively reduced by curcumin, DMC, and BDMC at 100 mg/kg bwt. This result aligns with the study of Alghadir et al., which found that oral curcumin supplementation significantly improved the damage caused by collagen-induced arthritis in rats [28]. No study is available regarding the effects of DMC and BDMC on the histological architecture of inflamed paw tissue. The curcuminoids may prevent the release of inflammatory mediators by inhibiting the COX-1 and COX-2 pathways, thereby preventing edema and tissue damage caused by carrageenan.

The compound, which has good antimicrobial and anti-inflammatory properties, showed excellent healing potential by preventing infection and inflammation at the wound site, thereby speeding up the healing process [29]. Since ancient times, people have regularly used turmeric to cure wounds and inflammation. Turmeric extract has been reported to be antimicrobial [30] and antifungal [2,3]. The crude extracts of turmeric and curcumin have been reported to have antimicrobial and anti-inflammatory effects [3,7,31]. However, research regarding the wound-healing properties of turmeric’s active components is insufficient. Most wound-healing studies have focused mainly on curcumin. However, we observed that all three curcuminoids have vigorous wound-healing activity, with no significant differences. This may be because the topical application makes them equally accessible at the wounded site. It has been reported that biodegradable polymeric films with entrapped DMC and BDMC enhance wound healing and attenuate inflammation efficacy [32]. Topical application or wound dressing with *Curcuma longa* extract gel or curcumin demonstrated efficacy both in normal and diabetic-impaired wounds in many studies [33,34,35]. In addition to being antibacterial, the antifungal, antioxidative, and anti-inflammatory properties make curcumin a potent healing agent by accelerating tissue regeneration and remodeling [36]. Delayed wound healing and excess inflammation are the complications of diabetes. Our compounds showed anti-inflammatory and wound-healing effects, so we further studied their anti-diabetic properties.

All the curcuminoids showed anti-diabetic effects, which were observed by their efficacy in improving body weight and decreasing blood glucose along with ALT and AST levels. BDMC was more potent than curcumin and DMC. Our results partially agree with those of Arun et al., who reported that crude extracts of turmeric and curcuminoids reduced blood glucose and glucose metabolic enzymes in streptozotocin-induced diabetes in rats [37,38]. They also found a better glucose-lowering effect of curcumin than of turmeric. Natural and synthetic curcumin analogs inhibit human pancreatic α-amylases and α-glucosidase [39]. Curcuminoids and sesquiterpenoids extracted from *Curcuma longa* demonstrated a strong hypoglycemic effect in KK-Ay mice with type 2 diabetes [14]. The α-glucosidase inhibitory activities are used to assess the anti-diabetic properties of a compound. According to Kalaycıoğlu et al. BDMC has been reported to have stronger α-glucosidase inhibitory characteristics than curcumin and DMC [40]. These reports support our findings that BDMC exhibited more potent anti-diabetic effects. A diet supplemented with 1% curcumin for 3 weeks significantly increased the plasma insulin level in type 1 diabetic rats [41]. ALT and AST are important hepatic enzymes, and their levels in the blood are helpful indicators of hepatic cellular integrity [42]. We observed that streptozotocin-induced diabetes increased ALT and AST levels in the serum, which were significantly inhibited by three turmeric active compounds. It has been reported that curcumin enhances the development of hepatic microvessels in low doses and repairs hepatocellular damage in diabetic rats [43]. BDMC has shown superior potentiality to curcumin and DMC in reducing obesity and related chronic inflammation [44]. Additionally, they discovered that the extremely low concentration of BDMC in turmeric extract was the reason for its minimal anti-adipogenic efficacy. Consequently, they propose that for significant anti-adipogenic efficacy, it is crucial to use turmeric cultivars with high BDMC concentrations [44]. Because the Ryudai gold cultivar of turmeric contains higher quantities of all three curcuminoids, it may be a good source of functional dietary supplements for DMC and BDMC. Despite diverse biological activities, the main limitation of curcumin is its poor oral bioavailability. Hence, more bioavailable DMC and BDMC could be promising alternatives in treating diabetes and its chronic complications like inflammation and wounds.

## 4. Materials and Methods

### 4.1. Experimental Animals

All the experimental animals (rats and mice) were collected from a reputed global health research institute (International Center of Diarrheal Disease Research, Dhaka, Bangladesh). The animals were acclimated to new environmental conditions for one week and then housed in different compartmentalized rectangular plastic cages according to the group. The cages were maintained in an adequately ventilated room (temperature 28 ± 2 °C, humidity 70–80%) with a natural daylight cycle and given full access to food and water. The pellet feed was prepared according to the recommendations of the National Research Council. The average regular feed consumption of every group was measured previously. All the experiments were carried out following the guidelines of Bangladesh Agricultural University’s Animal Welfare and Ethics Committee (AWEEC/BAU/2020(2)).

### 4.2. Active Compounds of Turmeric and Chemicals

We have previously isolated, purified, and studied the biological activities of some pure active compounds from the Ryudai gold variety of *Curcuma longa* [3,4]. This variety was registered by the Ministry of Agriculture, Forestry, and Fisheries, Japan (Registration No. 21485, 29 February 2012). DMC and BDMC are the main identified chemicals with good biological activity, along with curcumin. Thus, we looked into how these three curcuminoids, which we had isolated from our previous study, affected diabetes, wound healing, and inflammation. Carrageenan was purchased from Wako Pure Chemical Corporation (Osaka, Japan). Streptozotocin (Sigma-Aldrich, Saint Louis, MO, USA) was dissolved in a 0.1 M cold citrate buffer (pH 4.5). All additional chemicals and solvents used for the experiments were of analytical grade.

### 4.3. Anti-Inflammatory Study

The anti-inflammatory study was performed according to the methodology established by Kasahara et al. with minor modifications [45]. Male Wister rats were used in this experiment. Three groups of rats (n = 4) were treated with curcumin, DMC, and BDMC (100 mg/kg body weight) via oral gavage. The curcumin, DMC, and BDMC were dissolved in corn oil so that the oral gavage of 500 mL contained the required dose for a rat. An equal volume (500 mL) of corn oil was orally administered to the control group. Each rat was given an intraperitoneal injection of freshly prepared carrageenan suspension (1% in physiological saline; 50 µL) sixty minutes after the oral gavage of curcumin, DMC, and BDMC. The control paw was injected with an equal volume of saline. A digital slide caliper was used to measure the paw volume at one, three, and six hours following the development of inflammation. The edema percent was calculated using the following equation [46]:

Edema % = (*V*d − *V*c)/*V*c × 100%

where *V*d: paw volume of the inflamed paw and *V*c: paw volume of the control paw.

### 4.4. Histopathological Examination of the Paw Tissues

After 3 h of carrageenan injection, one rat from each group was euthanized to collect the paw tissue. The collected tissues were fixed in formalin (10%). Then, the tissues were dehydrated in a progressively higher concentration of alcohol, cleared in xylene, and embedded in paraffin wax. Hematoxylin and eosin were used to stain the 5 µm thick sections of paraffin blocks for microscopic examination.

### 4.5. Wound-Healing Study

In this experiment, twelve male Wister rats were utilized. Animals were evenly grouped into four groups: control, curcumin, DMC, and BDMC. Ketamine hydrochloride injections (2 mg/kg bwt) were used to anesthetize the animals, and diethyl ether inhalations were used to sustain them when needed. The dorsum of each mouse was shaved and disinfected. A biopsy punch (Kai Medical, Gifu, Japan) was used to create a circular wound (6 mm). Wounds were left open and treated with curcumin (1%), DMC (1%), and BDMC (1%). The curcuminoids were suspended in 0.5% sodium carboxymethyl cellulose and distilled water. This suspension was mixed vigorously with a mortar and pestle and applied topically twice daily with the help of a cotton brush until the wound was completely healed. Feed and water were supplied ad libitum to all groups. On days 0, 3, 6, 9, and 12, the wound sites were measured with a permanent marker on translucent paper.

The wound closing rate was calculated by using the following formula:

(Area of original wound−Area of the remaining wound)/Area of original wound × 100.

### 4.6. Anti-Diabetic Study

After one week of acclimatization, 35 male Swiss albino mice (age: 6 weeks, body weight: 35 ± 2 g) were randomly divided into 5 groups: (1) control; (2) diabetes; (3) diabetes + curcumin; (4) diabetes + DMC; and (5) diabetes + BDMC. After overnight starvation, streptozotocin (50 mg/kg bwt, i.p.) was injected to induce diabetes. The mice were supplied with a regular diet and water ad libitum until day 5 of the streptozotocin injection. On the fifth day of the streptozotocin injection, body weight and blood glucose levels were recorded to confirm the onset of diabetes. During the acclimatization period, we measured each group’s daily feed intake for one week. We calculated each group’s average daily feed intake and body weight. The estimated amount of curcuminoids (100 mg/kg/day body weight) for a group was incorporated with half of the required feed for that particular group for one week. We provided feed twice a day (7 a.m. and 7 p.m.). First, we offered curcuminoid-incorporated feed, and then, after finishing the mixed feed, we provided the basal diet. A small volume of blood was collected by puncturing the tail vein at a fixed time of the day (8:30–9:00 a.m.), and blood glucose was measured by a glucometer (GlucoLeader^TM^ Enhance, HMD BioMedical Inc., Hsinchu, Taiwan) every week for four weeks.

#### 4.6.1. Serum Biomarkers

After 4 weeks of treatment, ketamine hydrochloride injections (2 mg/kg bwt) were used to anesthetize the animals, and diethyl ether inhalations were used to sustain them when needed. Whole blood was obtained in a non-heparinized syringe by puncturing the thoracic vein. The blood was left undisturbed at room temperature for thirty minutes to allow it to clot. The drawn blood was centrifuged for 10 min at 2000 rpm to extract the serum. An autoanalyzer (Dimension^®^ RXL MAXTM, Siemens, Malvern, PA, USA) was used to assess the activity of the serum biomarker enzymes, such as ALT and AST.

#### 4.6.2. Liver Histology

After blood collection, the liver was removed and preserved in 10% formalin. Then, the liver tissue was dehydrated in a progressively higher concentration of alcohol, cleared in xylene, and embedded in paraffin wax. Hematoxylin and eosin were used to stain the 5 µm thick sections of paraffin blocks for microscopic examination.

#### 4.6.3. Statistical Analysis

The data were calculated as the mean ± SD (standard deviation). A post-hoc least significant difference (LSD) test was conducted after a one-way analysis of variance (ANOVA) was used to examine the variations between the groups. The level of statistical significance was set at *p* < 0.05. Statistical tests were not performed for histological experiments as they were considered nonparametric.

## 5. Conclusions

In this study, we aimed to compare the anti-inflammatory, wound healing, and anti-diabetic properties of DMC and BDMC with those of curcumin in animal models. Curcumin has been extensively studied for its diverse pharmacological activities. However, poor bioavailability is the primary constraint on its therapeutic use. DMC and BDMC are slightly different in chemical structure from curcumin, which could make them more bioavailable. However, information regarding their biological activities is scarce in comparison to curcumin. This might be because they are present in a very low concentration in turmeric compared to curcumin. As reported earlier, Ryudai gold contains a comparatively higher concentration of all three curcuminoids [2] and better antifungal and antioxidant activities than other turmeric varieties [3,4]. Therefore, this turmeric variety (Ryudai gold) could be an important functional food for lifestyle diseases like inflammation, diabetes, and wound-healing complications. These compounds could be the source of alternative natural drugs or natural-product-based lead molecules for synthesizing new drugs or analogs. However, more research is required to fully understand the mechanism by which curcuminoids produce these biological effects, and it is necessary to study the concentration–response curve of each molecule and compare them with those of control drugs.

## Data Availability

The data supporting this study’s findings are available from the corresponding author.
